# Propofol-based total intravenous anesthesia did not improve survival compared to desflurane anesthesia in breast cancer surgery

**DOI:** 10.1371/journal.pone.0224728

**Published:** 2019-11-07

**Authors:** Yi-Hsuan Huang, Meei-Shyuan Lee, Yu-Sheng Lou, Hou-Chuan Lai, Jyh-Cherng Yu, Chueng-He Lu, Chih-Shung Wong, Zhi-Fu Wu

**Affiliations:** 1 Department of Anesthesiology, Tri-Service General Hospital and National Defense Medical Center, Taipei, Taiwan, Republic of China; 2 School of Public Health, National Defense Medical Center, Taipei, Taiwan, Republic of China; 3 Division of General Surgery, Department of Surgery, Tri-Services General Hospital, National Defense Medical Center, Taipei, Taiwan, Republic of China; 4 Division of Anesthesiology, Cathay General Hospital, Taipei, Taiwan, Republic of China; 5 Department of Anesthesiology, Chi Mei Medical Center, Tainan City, Taiwan, Republic of China; China Medical University Hospital, TAIWAN

## Abstract

**Background:**

Breast cancer is the most common cancer in women and several perioperative factors may account for tumor recurrence and metastasis. The anesthetic agents employed during cancer surgery might play a crucial role in cancer cell survival and patient outcomes. We conducted a retrospective cohort study to investigate the relationship between the type of anesthesia and overall survival in patients who underwent breast cancer surgery performed by one experienced surgeon.

**Methods:**

All patients who underwent breast cancer surgery by an experienced surgeon between January 2006 and December 2010 were included in this study. Patients were separated into two groups according to the use of desflurane or propofol anesthesia during surgery. Locoregional recurrence and overall survival rates were assessed for the two groups (desflurane or propofol anesthesia). Univariable and multivariable Cox regression models and propensity score matching analyses were used to compare the hazard ratios for death and adjust for potential confounders (age, body mass index, American Society of Anesthesiologists physical status classification, TNM stage, neoadjuvant chemotherapy, Charlson Comorbidity Index, anesthesiologists, and functional status).

**Results:**

Of the 976 breast cancer patients, 632 patients underwent breast cancer surgery with desflurane anesthesia, while 344 received propofol anesthesia. After propensity scoring, 592 patients remained in the desflurane group and 296 patients in the propofol group. The mortality rate was similar in the desflurane (38 deaths, 4%) and propofol (22 deaths, 4%; *p* = 0.812) groups in 5-year follow-up. The crude hazard ratio (HR) for all patients was 1.13 (95% confidence interval [CI] 0.67–1.92, *p* = 0.646). No significant difference in the locoregional recurrence or overall 5-year survival rates were found after breast surgery using desflurane or propofol anesthesia (*p* = 0.454). Propensity score-matched analyses demonstrated similar outcomes in both groups. Patients who received propofol anesthesia had a higher mortality rate than those who received desflurane anesthesia in the matched groups (7% vs 6%, respectively) without significant difference (*p* = 0.561). In the propensity score-matched analyses, univariable analysis showed an insignificant finding (HR = 1.23, 95% CI 0.72–2.11, *p* = 0.449). After adjustment for the time since the earliest included patient, the HR remained insignificant (HR = 1.23, 95% CI 0.70–2.16, *p* = 0.475).

**Conclusion:**

In our non-randomized retrospective analysis, neither propofol nor desflurane anesthesia for breast cancer surgery by an experienced surgeon can affect patient prognosis and survival. The influence of propofol anesthesia on breast cancer outcome requires further investigation.

## Introduction

Breast cancer is one of the most common malignancies that affect women globally [1]. According to GLOBOCAN 2012, breast cancer is the leading cause of cancer-related deaths. Although the prevention of risk factors, early diagnostic screening, and advances in treatment [2] have improved cancer mortality rates, several perioperative factors may account for recurrence and metastasis, including the selection of anesthetic agents, perioperative regional analgesics, intraoperative opioids and nonsteroidal anti-inflammatory drugs(NSAIDs)/ cyclo-oxygenase (COX) inhibitors, surgical manipulation, and perioperative immunosuppression induced by surgical stress [3].

Recent reports discussed how anesthetics can influence cancer cell survival and progression [3, 4]. An old experimental study revealed that the use of halothane during surgical excision of local tumors strongly accelerated postoperative progression of spontaneous lung metastases produced by the 3LL Lewis lung carcinoma and by the B16 melanoma. Halothane induced the appearance of metastases in organs, such as the liver, in which spontaneous metastases were not usually produced by these tumors [5]. Benzonana *et al*. [6] reported that isoflurane upregulated the levels of hypoxia-inducible factor (HIF)-1α and HIF-2α and intensified the expression of vascular endothelial growth factor A in renal cell carcinoma cells. In a review article, Tavare *et al*. [7] concluded that halothane, isoflurane, and sevoflurane upregulated HIF genes in tumor cells resulting in poor prognosis. On the other hand, propofol reduced the levels of HIF-1α protein and was found to reduce the invasion and migration of breast cancer cells (MDA-MB-231) via inhibition of the NF-κB pathway [8]. Melamed *et al*. [9] demonstrated that propofol did not suppress natural killer (NK) cell activity or promote tumor metastasis in a rat model of breast cancer cells with pulmonary metastasis. Additionally, Kushida *et al*. [10] reported that propofol suppressed lymphoblast tumor growth in mice, suggesting that propofol enhances anti-tumor immunity. Another study reported that serum from patients who received sevoflurane anesthesia and opioids for primary breast cancer surgery exhibited attenuated apoptosis in estrogen receptor (ER)-negative breast cancer cells compared to serum from patients who received propofol-paravertebral anesthesia [11].

Previous retrospective studies analysed anesthetic type in breast cancer surgery [12–14] and found no association between volatile inhalation and propofol anesthesia with regard to the recurrence-free survival and overall survival of breast cancer, except Lee *at al*. [12] who suggest propofol-based anesthesia can lower the risk of breast cancer recurrence during the initial 5 years after surgery. However, these studies did not mention that surgeons might be one of the predictors of breast cancer outcome which would be regarded as one of the confounding factors. Chen *et al*. [15] analysed a pooled population-based database of the 13,360 breast cancer surgery patients and concluded that high surgeon volume is significantly associated with positive patient outcomes in Taiwan.

To the best of our knowledge, there has been limited research on the effects of one of the inhalation agents, desflurane, and one surgeon on breast cancer in vivo. Thus, we conducted a single center retrospective cohort study to assess whether the choice of anesthetics, volatile inhalation agent, desflurane, and propofol anesthesia affects recurrence and overall 5-year survival in patients that underwent breast cancer surgery performed by one experienced surgeon which the surgeon-related confounding could be excluded.

## Materials and methods

### Study design

This was a retrospective cohort study.

### Setting

This study was conducted at the Tri-Service General Hospital (Taipei, Taiwan, Republic of China).

### Participants and data sources

**Fig 1:**
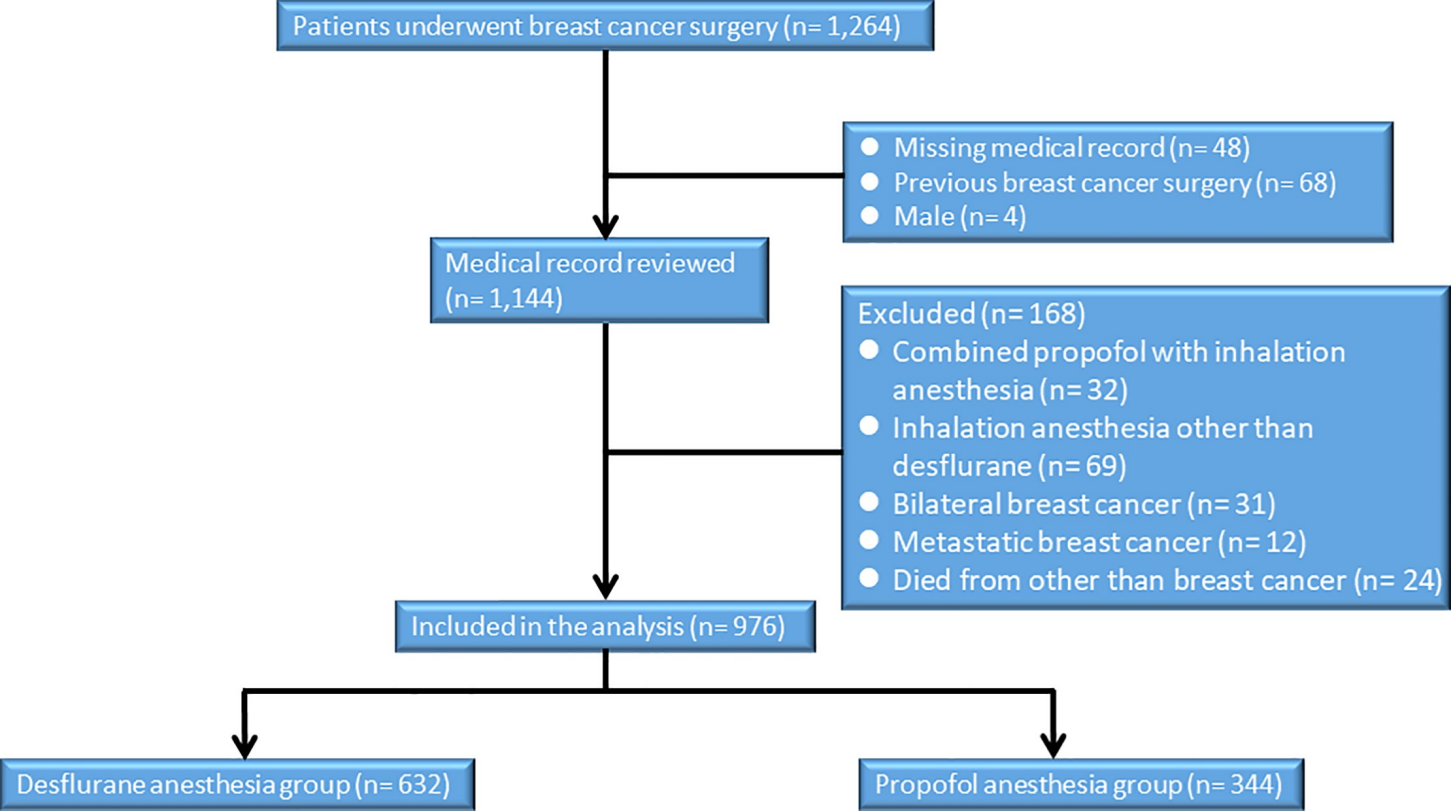
Flow diagram of the study population.

After approval from the ethics committee (TSGHIRB No: 1-104-05-139) of the Tri-Service General Hospital (TSGH), Taipei, Taiwan, Republic of China, relevant information was retrieved from the medical records and the electronic database of TSGH and the requirement for written informed consent was waived by the IRB. This retrospective study included 976 patients treated from January 2006 to December 2010. The patients had an American Society of Anesthesiologists (ASA) score of I–III and had undergone breast cancer surgery by an experienced surgeon (JC Yu) for tumor–node–metastasis (TNM) stage I–IV breast cancer. Six hundred and thirty-two patients were subjected to desflurane anesthesia and 344 underwent surgery under the influence of propofol anesthesia. No combination of propofol and inhalation anesthesia, isoflurane, or sevoflurane was used with our patients. Two hundred and eighty-eight patients were excluded from the analysis. The exclusion criteria were the use of propofol combined with inhalation anesthesia or inhalation agents other than desflurane, missing medical records, bilateral breast cancer, previous breast cancer surgery, metastatic breast cancer, death from other diseases, male gender or age < 20 years. (Fig 1)

All breast cancer surgery was performed by the same surgeon and no prior medications were prescribed before the induction of anesthesia. Hemodynamic monitoring, including non-invasive blood pressure, electrocardiography (lead II), pulse oximetry, and end-tidal carbon dioxide pressure (EtCO_2_) were performed. The selection of anesthetics was at the discretion of the attending anesthesiologist. Anesthesia was induced with fentanyl (2 μg kg^–1^), lidocaine (2%, 1.5 mg kg^–1^), propofol (2–3 mg kg^–1^), rocuronium (0.6 mg kg^–1^), or cisatracurium (0.1–0.2 mg kg^–1^) combined with desflurane in the desflurane group. In the propofol group, anesthesia was induced with fentanyl (2 μg kg^–1^), lidocaine (2%, 1.5 mg kg^–1^), a target-controlled infusion (TCI, Fresenius Orchestra Primea; Fresenius Kabi AG, Bad Homburg, Germany) with propofol Ce 4–5 μg mL^–1^ and rocuronium (0.6 mg kg^–1^) or cisatracurium (0.1–0.2 mg kg^–1^). Then, the patients were intubated and maintained with either propofol or desflurane, as well as the analgesic fentanyl. The following data were collected for each patient: gender, age, ASA score, the anesthesiologist, TNM stage, radiotherapy, chemotherapy, hormone therapy, and vital status.

In the desflurane group, anesthesia was maintained with 8–12% desflurane under a 100% oxygen flow of 300 mL min^–1^ in a closed system. In the propofol group, anesthesia was maintained with propofol Ce 3–4 μg mL^–1^ and an oxygen flow of 300 mL min^–1^ with 100% FiO_2_. Bolus injections of muscle relaxants (rocuronium or cisatracurium) and fentanyl were administered repeatedly as required throughout the procedure.

The anesthesiologists adjusted the Ce using TCI with propofol or desflurane at a range of 0.2–0.5 μg mL^–1^ or 0.5–2% for maintenance based on hemodynamic changes. The ventilation rate and maximum airway pressure were modulated to maintain EtCO_2_ at a range between 35–45 mmHg. Bolus dosing of either rocuronium (10 mg) or cisatracurium (2 mg) was prescribed intravenously as required during the recovery of neuromuscular function. During skin closure, desflurane or propofol was discontinued and patients were ventilated with 100% oxygen at a fresh gas flow rate of 6 L min^–1^. When the patient regained consciousness with spontaneous and smooth breathing, the endotracheal tube was removed. Then the patients were transferred to the post-anesthetic care unit for further care [16, 17].

### Variables

Patient data was obtained from the medical records and the electronic database including age at the time of surgery, body mass index (BMI), ASA score, TNM stage, neoadjuvant chemotherapy, the Charlson Comorbidity Index (CCI), anesthesiologists, preoperative functional status regarding metabolic equivalents (METs) to evaluate preoperative cardiorespiratory function and potentially predict perioperative outcomes, histologic grade, ER status, progesterone receptor (PR) status, epidermal growth factor receptor type 2 (HER-2) expression, tri-negative breast cancer (TNBC), and postoperative adjuvant hormonal therapy, chemotherapy or radiotherapy. In addition, we recorded the use of perioperative or postoperative opioids and NSAIDs, the duration of surgery and anesthesia, and the time of first metastasis.

### Study sample size

To achieve a power of 80% and a two-tailed type I error rate of α = 0.05, each unmatched group required 213 patients (assuming a mortality rate of 24% in the desflurane anesthesia group and 13.5% in the propofol anesthesia group). Each matched group required 465 patients (assuming a mortality rate of 22.8% in the desflurane anesthesia group and 15.6% in the propofol anesthesia group) [18].

### Statistical analysis

The major goal of our study was to identify the influences of different anesthetic agents (desflurane and propofol) on cancer recurrence and overall survival follow-up for 5 years after the surgery. Clinical evidence of locoregional recurrence or distant metastases confirmed by imaging studies or tissue-proved was defined as recurrence. Recurrence-free survival was defined from the date of operation to the date of first recurrence, death due to breast cancer, or the last follow-up, whichever occurred first. Overall survival was defined from the interval between the date of surgery and the date of the final outcome, distant metastasis, or end of follow-up in January 2016.

Patient characteristics and overall survival rates were compared between different anesthetics using the chi-square test, Fisher exact test or Student’s *t*-test. A propensity score (PS) was constructed to address the differences in baseline characteristics [19] between the two groups using a linear (simple logistic regression) algorithm. Interaction terms did not improve the model fit. In our observational studies, the influence of anesthetic effect on breast cancer outcome may be biased with nonrandomly allocate exposure. To dealing with confounding, an alternative approach, propensity score method is used [19, 20]. In order to minimizing confounding, matching algorithms is prescribed to find best matches between both groups. Since the desflurane group contained more patients than the propofol group did, to maximize statistical power, a greedy nearest-neighbor matching procedure with calipers set at 0.2 SD of the logit of the PS was used to create 1-to-2 matched pairs (296 pairs). The relationship between the choice of anesthetic (desflurane or propofol) and survival was analyzed using the Cox proportional-hazards model and PS-matching with adjustments for age, BMI, ASA physical status classification, anesthesiologists, TNM stage, neoadjuvant chemotherapy, CCI, and preoperative functional status. R (version 3.4.3, available at https://cran-r-project.org/src/base/r-3/r-3.4.3.tar.gz) and SPSS v22 were used for statistical analyses. *P*-values <0.05 were considered significant.

## Results

We reviewed 1,264 breast cancer patients who underwent breast cancer surgery, among which 632 received desflurane and 344 received propofol. Table 1 shows the patient baseline characteristics and treatment. The time since the earliest included patients was significantly longer in the propofol group (3.4 ± 1.2 years) than in desflurane group (2.2 ± 1.4 years; *p* < 0.001). The patient demographics for the two groups of both overall and matched patients, including age and BMI, were not statistically different. Prognostic factors, such as TNM stage, neoadjuvant chemotherapy, postoperative adjuvant chemotherapy, radiotherapy, hormonal therapy of breast cancer and intraoperative NSAIDs, HER-2 expression, ER expression, PR expression and TNBC were all similar in the desflurane and propofol groups of overall and matched patients. The propofol group of overall patients had significantly more patients with ASA scores ≥ II (*p* = 0.009) than the desflurane group did, but there was no significant difference with ASA scores ≥ II (*p* = 0.077) in both groups after matching. The CCI score was significantly higher in the propofol group than in the desflurane group (*p* = 0.008) in overall patients, but was insignificant after propensity scoring (*p* = 0.318). More patients in the propofol group received postoperative NSAIDs (*p* = 0.039); nevertheless, there was no statistical significance in the both groups after matching (*p* = 0.067). The presence of local recurrence revealed no significant differences between the desflurane group (4%) and the propofol group (4%). Besides, the percentage of patients with distant metastases in the desflurane group (8%) was higher than in the propofol group (6%). But the difference was not statistically significant both in all patients and matched patients (*p* = 0.454 *vs*. *p* = 0.707). The mortality rate was similar in the desflurane (38 deaths, 6%) and propofol (22 deaths, 6%; *p* = 0.812) groups in overall patients and the finding was also similar in the matched patients (*p* = 0.561).

**Table 1:** Patient and treatment characteristics for overall patients and matched patients after propensity scoring.

	Overall patients			Matched patients			
Variable	Desflurane N = 632	Propofol N = 344	p-value	Desflurane N = 592	Propofol N = 296	*p*-value	SMD
Time since the earliest included patient (yr)	2.2 ± 1.4	3.4 ± 1.2	< 0.001	2.2 ± 1.4	3.3 ± 1.2	< 0.001	0.888
Age (yr)			0.512			0.542	0.081
< 40	64 (10)	32 (9)		58 (10)	27 (9)		
40–49	245 (39)	117 (34)		229 (39)	104 (35)		
50–59	212 (34)	126 (37)		203 (34)	111 (38)		
60–69	82 (13)	48 (14)		76 (13)	35 (12)		
≥ 70	29 (5)	21 (6)		26 (4)	19 (6)		
BMI (kg/ m^2^)	23.3 ± 3.5	23.3 ± 3.6	0.709	23.3 ± 3.4	23.1 ± 3.6	0.397	0.060
ASA			0.009			0.077	0.157
I	428 (68)	199 (58)		403 (68)	179 (61)		
II	175 (28)	123 (36)		162 (27)	99 (33)		
III	29 (5)	22 (6)		27 (5)	18 (6)		
Functional status			0.206			0.180	0.096
≥ 4 METs	605 (96)	323 (94)		568 (96)	278 (94)		
< 4 METs	27 (4)	21 (6)		24 (4)	18 (6)		
CCI			0.008			0.318	0.130
2	474 (75)	228 (66)		445 (75)	207 (70)		
3	108 (17)	67 (20)		102 (17)	59 (20)		
4	37 (6)	37 (11)		36 (6)	22 (7)		
≥ 5	13 (2)	12 (4)		9 (2)	8 (3)		
TNM stage of primary tumor			0.995			0.578	0.009
0	108 (17)	58 (17)		100 (17)	46 (16)		
I	235 (37)	130 (38)		215 (36)	120 (41)		
II	210 (33)	112 (33)		203 (34)	91 (31)		
III	79 (13)	44 (13)		74 (13)	39 (13)		
HER-2, Negative	301 (48)	184 (54)	0.080	282 (48)	156 (53)	0.154	0.102
ER, Negative	203 (32)	88 (26)	0.039	192 (32)	78 (26)	0.075	0.133
PR, Negative	136 (22)	66 (19)	0.450	127 (22)	57 (19)	0.516	0.053
TNBC	30 (5)	14 (4)	0.745	28 (5)	13 (4)	0.955	0.043
Neoadjuvant chemotherapy	29(5)	16(5)	0.964	25(4)	15(5)	0.567	0.049
Intraoperative NSAIDs	33 (5)	14 (4)	0.422	31 (5)	11 (4)	0.314	0.072
Postoperative NSAIDs	13 (2)	15 (4)	0.039	13 (2)	13 (4)	0.067	0.131
Adjuvant chemotherapy	369 (58)	194 (56)	0.548	349 (59)	170 (57)	0.665	0.031
Adjuvant radiotherapy	348 (55)	167 (49)	0.051	326 (55)	143 (48)	0.057	0.136
Adjuvant hormonal therapy	473 (75)	254 (74)	0.731	442 (75)	215 (73)	0.516	0.046
Relapse			0.454			0.707	0.059
No	555 (88)	311 (90)		521 (88)	266 (90)		
Local recurrence in 5 years	28 (4)	13 (4)		25 (4)	11 (4)		
Distant metastases in 5 years	49 (8)	20 (6)		46 (8)	19 (6)		
Survival							
2006–2010	594 (94)	322 (94)	0.812	556 (94)	275 (93)	0.561	
2006	138 (92)	29 (94)	1.000	130 (92)	26 (93)	1.000	
2007	167 (95)	5 (100)	1.000	157 (95)	4 (100)	1.000	
2008	126 (91)	51 (98)	0.118	118 (92)	47 (98)	0.184	
2009	51 (94)	134 (91)	0.568	47 (94)	114 (90)	0.562	
2010	112 (98)	108 (95)	0.270	104 (98)	84 (94)	0.249	

Data shown as mean ± SD or n (%.)

BMI = body mass index; ASA = American Society of Anesthesiologists; TNM = tumor–node–metastasis; CCI = Charlson comorbidity index; MET = metabolic equivalents; NSAID = nonsteroidal anti–inflammatory drugs; TNBC = triple–negative breast cancer.

Before the surgery, we used the PS from logistic regression to adjust the baseline characteristics and the choice of therapy between the two groups. Since there were more patients in the desflurane group, 1-to-2 matched pairs (296 pairs) was formed to retain statistical power. The standardized mean differences (SMD) for the variables were not all < 0.1, such as time since the earliest included patient, the ASA score, CCI, negative HER-2, negative ER, postoperative NASIDs, and adjuvant radiotherapy.

Data was collected from January 2006 to December 2010 and fewer patients received propofol anesthesia from 2006 to 2008. The number of patients who received propofol increased over 5 years, and the survival rate for the two groups varied each year (Table 1). Therefore, time might be a confounding factor because changes in cancer care over time could have influenced the outcomes. After PS matching, the SMD of time since the earliest included patients remained > 0.1 (Table 1); therefore, matched and unmatched group analyses were adjusted for time since the earliest included patients to avoid any possible confounding effects due to the time factor.

Overall survival from the date of surgery grouped according to anesthesia type and other variables was compared separately in a univariable Cox model and subsequently in a multivariable Cox regression. Multivariable analyses revealed some variables related to the risk of death, including age, ASA score ≥ II, advanced TNM stage, positive ER, positive PR, neoadjuvant chemotherapy, adjuvant chemotherapy, and adjuvant hormonal therapy both in all patients and matched patients (Tables 2 and 3) and intraoperative NSAIDs in matched patients (Table 3). Patients who received propofol anesthesia had a higher mortality rate than those who received desflurane anesthesia in the matched groups (7% vs 6%, respectively) without significant difference (*p* = 0.561) (Table 1). The crude hazard ratio (HR) for all patients was 1.13 (95% confidence interval [CI] 0.67–1.92, *p* = 0.646). After adjustment for potential covariates, the HR became 1.17 (95% CI 0.68–2.00, *p* = 0.577) (Table 2). Similarly, in the propensity score-matched analyses, univariable analysis showed an insignificant finding (HR = 1.23, 95% CI 0.72–2.11, *p* = 0.449). The adjusted HR remained insignificant (HR = 1.23, 95% CI 0.70–2.16, *p* = 0.475) (Table 3).

**Table 2:** Cox regression proportional hazard survival: Univariable and multivariable models for overall patients (n = 976).

Independent variable	Crude-HR (95% CI)	*p*-value	Adj-HR (95% CI)	*p*-value
Anesthesia, propofol (ref: Desflurane)	1.13 (0.67–1.92)	0.646	1.17 (0.68–2.00)	0.577
Time since the earliest included patient (yr) (ref: < 40)	0.94 (0.78–1.12)	0.473		
Age (yr)				
40–49	0.32 (0.17–0.73)	0.005	0.45 (0.21–0.94)	0.034
50–59	0.49 (0.24–0.98)	0.045	0.50 (0.24–1.03)	0.061
60–69	0.23 (0.08–0.72)	0.011	0.15 (0.05–0.51)	0.002
≥70	0.82 (0.29–2.32)	0.701	0.43 (0.13–1.44)	0.171
BMI (kg/ m^2^)	1.04 (0.97–1.11)	0.281		
ASA (ref: I)				
II	1.74 (1.01–3.01)	0.047	0.80 (0.40–1.58)	0.523
III	3.69 (1.69–8.09)	0.001	3.55 (1.38–9.15)	0.009
Functional status, < 4 METs (ref: ≥ 4 METs)	2.25 (0.97–5.23)	0.06		
CCI (ref: 2)				
3	0.61 (0.28–1.35)	0.225		
4	1.58 (0.71–3.51)	0.260		
≥ 5	0.65 (0.09–4.72)	0.672		
TNM Stage of primary tumor, II + III (ref: 0 + I)				
II + III	5.44 (2.58–11.44)	< 0.001	6.82 (2.96–15.7)	< 0.001
HER-2 (ref: negative)	1.28 (0.77–2.14)	0.339		
ER (ref: negative)	0.45 (0.27–0.74)	0.002	0.62 (0.33–1.17)	0.138
PR (ref: negative)	0.56 (0.33–0.97)	0.038	1.18 (0.61–2.28)	0.626
TNBC (ref: no)	0.35 (0.05–2.50)	0.294		
Neoadjuvant chemotherapy (ref: no)	13.5 (7.86–23.1)	< 0.001	10.5 (5.36–20.7)	< 0.001
Intraoperative NSAIDs (ref: no)	2.26 (0.97–5.26)	0.059		
Postoperative NSAIDs (ref: no)	1.14 (0.28–4.66)	0.858		
Adjuvant chemotherapy (ref: no)	3.14 (1.63–6.03)	< 0.001	0.71 (0.32–1.60)	0.414
Adjuvant radiotherapy (ref: no)	0.95 (0.57–1.58)	0.848		
Adjuvant hormonal therapy (ref: no)	0.36 (0.22–0.60)	< 0.001	0.50 (0.27–0.93)	0.028
Relapse (ref: no)	61.4 (29.1–129)	< 0.001		

All multivariable HRs were adjusted by those variables significant (*p* < 0.05) in the univariable analyses simultaneously except anesthesia. Relapse status was also excluded from the multivariable model because it was an intermediary variable in the cause path to outcome.

BMI = body mass index; ASA = American Society of Anesthesiologists; TNM = tumor–node–metastasis; CCI = Charlson comorbidity index; MET = metabolic equivalents; NSAID = nonsteroidal anti–inflammatory drugs; ER = estrogen receptor; PR = progesterone receptor; TNBC = triple–negative breast cancer.

**Table 3:** Cox regression proportional hazard survival: Univariable and multivariable models for matched patients (n = 888).

Independent variable	Crude-HR (95% CI)	*p*-value	Adj-HR (95% CI)	*p*-value
Anesthesia, propofol (ref: desflurane)	1.23 (0.72–2.11)	0.449	1.23 (0.70–2.16)	0.475
Time since the earliest included patient (yr) (ref: < 40)	0.95 (0.79–1.15)	0.617		
Age (yr)				
40–49	0.37 (0.17–0.79)	0.010	0.53 (0.24–1.14)	0.103
50–59	0.49 (0.24–1.01)	0.054	0.52 (0.24–1.10)	0.087
60–69	0.26 (0.08–0.81)	0.021	0.20 (0.06–0.68)	0.010
≥ 70	0.69 (0.22–2.18)	0.531	0.37 (0.10–1.41)	0.144
BMI (kg/ m^2^)	1.05 (0.98–1.13)	0.179		
ASA (ref: I)				
II	1.74 (0.99–3.06)	0.056	0.81 (0.39–1.65)	0.555
III	3.91 (1.78–8.59)	0.001	4.50 (1.73–11.7)	0.002
Functional status, < 4 METs (ref: ≥ 4 METs)	1.97 (0.79–4.94)	0.147		
CCI (ref: 2)				
3	0.45 (0.18–1.14)	0.092		
4	1.82 (0.82–4.05)	0.141		
≥ 5	0.87 (0.12–6.31)	0.890		
TNM Stage of primary tumor, II + III (ref: 0 + I)				
II + III	7.42 (3.52–15.68)	< 0.001	6.15 (2.67–14.1)	< 0.001
HER–2 (ref: negative)	1.23 (0.73–2.87)	0.444		
ER (ref: negative)	0.45 (0.27–0.75)	0.002	0.65 (0.34–1.16)	0.202
PR (ref: negative)	0.57 (0.33–1.00)	0.049	1.23 (0.61–2.45)	0.562
TNBC (ref: no)	0.36 (0.05–2.61)	0.313		
Neoadjuvant chemotherapy (ref: no)	14.8 (8.49–25.62)	< 0.001	11.4 (5.60–23.0)	< 0.001
Intraoperative NSAIDs (ref: no)	2.50 (1.07–5.82)	0.034	1.01 (0.40–2.54)	0.978
Postoperative NSAIDs (ref: no)	1.16 (0.28–4.77)	0.835		
Adjuvant chemotherapy (ref: no)	3.23 (1.63–6.39)	0.001	0.78 (0.34–1.79)	0.552
Adjuvant radiotherapy (ref: no)	1.00 (0.60–1.70)	0.991		
Adjuvant hormonal therapy (ref: no)	0.36 (0.21–0.60)	< 0.001	0.50 (0.26–0.94)	0.031
Relapse (ref: no)	45.8 (25.6–81.8)	< 0.001		

All multivariable HRs were adjusted by those variables significant (*p* < 0.05) in the univariable analyses simultaneously except anesthesia. Relapse status was also excluded from the multivariable model due to it was an intermediary variable in the cause path to outcome.

BMI = body mass index; ASA = American Society of Anesthesiologists; TNM = tumor–node–metastasis; CCI = Charlson comorbidity index; MET = metabolic equivalents; NSAID = nonsteroidal anti–inflammatory drugs; ER = estrogen receptor; PR = progesterone receptor; TNBC = triple–negative breast cancer.

The distributions of types of anesthesia among total 20 anesthesiologists were significantly different both in all patients and matched patients (*p* < 0.001; S2 Table). Nevertheless, after adjustment for anesthesiologists, the adjusted HR for all patients was 0.70 (95% CI 0.31–1.56, *p* = 0.383) and the adjusted HR for matched patients was 0.67 (95% CI 0.28–1.61, p = 0.369) (S3 Table).

We also found that relapse, ASA scores ≥ III, advanced TNM stage and neoadjuvant chemotherapy contributed to higher mortality. Additionally, age > 40 years and adjuvant hormonal therapy improved survival. (Tables 2 and 3)

## Discussion

Our retrospective study demonstrated that propofol and desflurane anesthesia were not associated with overall survival following breast cancer surgery by an experienced surgeon during the initial 5-year follow-up. We found that neither agent had a significant effect on survival rate, metastasis, or recurrence.

Lee *et al*. [12] suggested that propofol-based anesthesia for breast cancer surgery attenuates the risk of cancer recurrence, but does not improve survival rate during the initial 5 years when compared to sevoflurane-based anesthesia. Kim *et al*. [13] found there was no difference in breast cancer recurrence between total intravenous anesthesia (2% propofol and remifentanil) and balanced anesthesia (sevoflurane, desflurane, isoflurane, or enflurane with adjuvant intravenous infusion of remifentanil). Yoo *et al*. [14] compared total intravenous anesthesia with inhalation anesthesia (sevoflurane, desflurane, enflurane, or isoflurane) which also revealed no association between anesthetic type and recurrence-free survival or overall survival. In addition, Wigmore *et al*. [18] reported that the breast cancer mortality with isoflurane or sevoflurane anesthesia was 8.6% (52/603) while that with propofol anesthesia was 6.6% (103/1560). However, Enlund *et al*. [21] found that the difference in survival (propofol minus sevoflurane anesthesia) after breast cancer surgery was 0.03 (95% CI 0.01–0.04, *p* < 0.001) in one-year and 0.02 (95% CI -0.02–0.06, data was not significant) in five years.

Soltanizadeh *et al*. [22] conducted a systemic review of the outcomes of cancer surgery with inhalational and intravenous anesthesia and concluded that propofol might be the optimal anesthetic choice. In addition, we demonstrated the use of propofol-based anesthesia for colon cancer surgery resulted in better survival than desflurane anesthesia irrespective of TNM stage [23]. These studies suggest an anti-tumor role for propofol [18, 22], but our findings are not in agreement with this hypothesis.

Woo *et al*. [24] investigated whether desflurane and propofol anesthesia application during breast cancer surgery preserved interleukin (IL)-2/IL-4 and the cluster of differentiation (CD)4(+)/CD8(+) T cell ratio with a favorable immune response. However, the study did not include long-term follow-up for the outcomes of the cancers, or the interactions between the immune system and surrounding factors. Interestingly, we compared propofol with desflurane anesthesia instead of sevoflurane. Nevertheless, the two drugs showed no difference in the survival rate and metastases in breast cancer patients.

Previous research revealed that volatile agents, such as halothane, isoflurane and sevoflurane, inhibited interferon α/β stimulated NK cell cytotoxicity and promoted apoptosis in human T lymphocytes in vivo and in vitro, resulting in a deleterious effect on tumor metastasis [9, 25–28]. Volatile anesthetics act via specific cell signalling mechanisms such as HIF-1α [6, 29, 30] leading to the accommodation and survival of healthy cells. A systemic review revealed that volatile anesthetics can induce tumor dissemination in animal models [31]. Therefore, compared to propofol, volatile agents are less preferable as anesthetics in cancer surgery [12, 21, 22].

Propofol attenuates tumor invasion and dissemination by reducing the expression of matrix metalloproteinases (MMPs) through the inhibition of NF-κB [8]. MMPs are the key enzymes in the breakdown of the basement membrane, and are therefore involved in oncologic outcomes. Experiments showed that propofol induces apoptosis in breast cancer cells by suppressing the miR-24/p27 signal pathway [32] and Kras mutation in breast cancer cells may play a role in propofol-induced apoptosis [33]. However, Meng *et al*. [34] reported that propofol increased the proliferation of human breast cancer MDA-MB-231 cells and induced cell migration. Despite reports of the anti-cancer effects and benefits of propofol in cancer surgery [12, 21, 22], our data did not support this conclusion.

Our study revealed neoadjuvant chemotherapy to be associated with poor survival after breast cancer surgery which was consistent with several studies of high risk of local recurrence or locoregional recurrence after breast cancer surgery [35–37]. In addition, we found age ≥ 40 years was associated with better survival than age < 40 years in agreement with a previous study that young age was an independent prognostic indicator for locoregional recurrence after breast cancer surgery [38]. Furthermore, we also found adjuvant hormonal therapy improved survival which may imply hormone replacement therapy with a beneficial effect on breast cancer outcome [39].

Surgery plays a crucial role in tumor metastasis during the perioperative period [40]. Manipulation of a tumor and its vasculature releases tumor cells into the host blood and lymphatic circulation, resulting in distant metastasis [41]. Local and systemic release of growth factors and reduced anti-angiogenic factors after surgery may induce the development of micro-metastasis and recurrence [41–43]. Moreover, surgery that induces the stress response can transiently suppress cell-mediated immunity [44–46] and eventually cause the spread of tumor cells. Oh *et al*. [47] concluded that the effect of anesthetics on the perioperative immune activity may be minimal during breast cancer surgery. Consequently, compared with the resection of other solid tumors, mastectomy was performed subcutaneously and caused less inflammatory reactions which may partially explain the difference between significant outcome with propofol-based anesthesia for colon cancer surgery in our previous study [23] and insignificant results for breast cancer surgery.

In our hospital, the average number of patients who had breast cancer and underwent breast cancer surgery by the specialist surgeon in the last 10 years was more than 350 annually. Sainsbury *et al*. [48] reported that the treatment strategy employed by surgeons with low caseloads reduced overall survival. A large retrospective population-based analysis by Stefoski-Mikeljevic and his colleagues [49] also showed the relative risk of death was lower for patients managed by surgeons with higher workloads. Moreover, Kingsmore *et al*. [50] investigated treatment by well-trained specialist surgeons was associated with half the risk of inadequate treatment of the breast cancer, a five-fold lower risk of inadequate axillary staging and nine times lower risk of inadequate definitive axillary treatment, 57% lower local recurrence rates at eight years, and 20% lower risk of death from breast cancer after allowing for case-mix and adjuvant therapies which implied adequate management of breast cancer surgery is essential to ameliorating the prognosis of breast cancer. Skinner *et al*. [51] also showed surgeons who performed more than 15 breast cancer surgeries per year achieved better 5-year survival than whom performed 1 to 5 breast cancer surgeries per year. A large retrospective study was reported by Chang *et al*. which analyzed outcomes of 77,971 patients after breast cancer surgery revealed that breast cancer outcomes were significant associated with surgeon seniority and volume in Taiwan [52]. Since all patients with breast cancer received surgery that was performed by an experienced surgeon in our study, the generalizability may not be guaranteed. However, this restriction can minimize the impact of different surgeons on outcome of different anesthetic techniques. The possible explainations on breast cancer outcome may be related to advanced surgical skill and appropriate use of adjuvant therapies. The major difference from the previous reports [12–14, 18, 21] is our multivariable analysis which showed no significant difference in the 5-year survival between desflurane and propofol anesthesia possibly because the patients were treated by an experienced specialist surgeon. Additionally, the low mortality rate of breast cancer may have interfered with our study results, leading to no significant difference in the survival rates for both groups [53]. Moreover, the previous reports in Asian patients with breast cancer were no significant survival [12–14] between anesthetics compared with Western patients with breast cancer [21]. Therefore, the ethnic effects might be considered.

Our study had a few limitations. First, this is a retrospective study. Patients were not randomly allocated and characteristics such as the ASA score and TNM stage may have introduced uncontrolled biases. Second, potential confounding factors and selection bias may exist due to the lack of perioperative anesthesia care standardization. Fifty percentage of the patients received total intravenous anesthesia performed by one anesthesiologist (Anesthesiologist A), therefore, these may cause some selection bias. However, we further adjusted for the effect of anesthesiologists, and figured out that the factor of anesthesiologists was not associated with breast cancer mortality (S3 Table) which was consistent with previous study that anesthesiologist volumes were not risk factors for postoperative mortality or long-term survival after radical cystectomy for bladder cancer in high volume hospital [54]. Third, we only analyzed perioperative factors with one experienced surgeon. Further medical treatment, oncologists, and radiation therapy, which are potential confounding factors, were varied. Fourth, we only investigated desflurane which was the most used volatile anesthetic in our hospital. Fifth, early screening is considered to increase the rate of diagnosis in breast cancer and are related to good prognosis of 5-year follow-up in early stage breast cancer. Long-term follow-up of > 5 years in breast cancer is taken into account to evaluate the difference of both groups. Sixth, this study was conducted in a single center. To investigate our hypothesis further, multicenter studies are required.

In conclusion, propofol and desflurane have no obvious differences in prognosis and survival after breast cancer surgery by an experienced surgeon. Further prospective studies should be conducted to identify the influence of propofol on breast cancer outcomes.

## Supporting information

S1 Table Disease history and conditions for overall patients and matched patients after propensity scoring. (DOCX)

S2 Table Distribution of anesthesiologist and risk of mortality for overall patients and matched patients after propensity scoring. (DOCX)

S3 Table Adjusted hazard ratio (HR) (95% CI) from the Cox regression proportional hazard survival models including anesthesiologists for overall patients (N = 976) and the matched patients (N = 888). (DOCX)
