# Supplementary material for: Propofol-based total intravenous anesthesia did not improve survival compared to desflurane anesthesia in breast cancer surgery
Source: PLoS One. 2019 Nov 7;14(11):e0224728. doi: 10.1371/journal.pone.0224728 (PMC6837387; doi:10.1371/journal.pone.0224728)
Supplement: S1 Table — (DOCX) [file pone.0224728.s001.docx]

**S1 Table.** Disease history and conditions for overall patients and matched patients after propensity scoring

|  | **Overall patients** | |  | **Matched patients** | |  |
| --- | --- | --- | --- | --- | --- | --- |
| **Diseases and condotions** | **Desflurane**  N = 632 | **Propofol**  N = 344 | ***p*-value** | **Desflurane**  N = 592 | **Propofol**  N = 296 | ***p*-value** |
| Hypertension | 103 (16) | 74 (22) | 0.053 | 96 (16) | 57 (19) | 0.300 |
| Diabetes | 29 (5) | 23 (7) | 0.213 | 24 (4) | 17 (6) | 0.336 |
| Stroke | 1 (0) | 4 (1) | 0.055 | 1 (0) | 3 (1) | 0.111 |
| Hyperlipidemia | 7 (1) | 2 (1) | 0.505 | 6 (1) | 2 (1) | 0.726 |
| Hyperthyroidism | 9 (1) | 6 (2) | 0.908 | 8 (1) | 5 (2) | 0.769 |
| Hypothyroidism | 3 (1) | 3 (1) | 0.431 | 3 (1) | 3 (1) | 0.406 |
| Ischemic heart disease | 19 (3) | 11 (3) | 1.000 | 17 (3) | 9 (3) | 1.000 |
| Valvular heart disease | 6 (1) | 8 (2) | 0.096 | 6 (1) | 8 (3) | 0.083 |
| COPD | 5 (1) | 4 (1) | 0.728 | 5 (1) | 3 (1) | 1.000 |
| Af | 4 (1) | 1 (0) | 0.662 | 3 (1) | 1 (0) | 1.000 |
| Alzheimer's disease | 0 | 0 |  | 0 | 0 |  |
| Parkinsonism | 1 (0) | 0 (0) | 1.000 | 0 | 0 |  |
| Mental disorder | 17 (3) | 6 (2) | 0.478 | 15 (3) | 5 (2) | 0.576 |
| Pregnancy | 2 (0) | 1 (0) | 1.000 | 2 (0) | 1 (0) | 1.000 |
| HBV/HCV carrier | 14 (2) | 14 (4) | 0.145 | 13 (2) | 6 (2) | 1.000 |
| Rheumatic disease | 3 (1) | 6 (2) | 0.074 | 3 (1) | 5 (2) | 0.125 |
| CKD/ renal dialysis | 3 (1) | 1 (0) | 1.000 | 3 (1) | 1 (0) | 1.000 |
| Previous malignancy | 6 (1) | 9 (3) | 0.080 | 6 (1) | 5 (2) | 0.521 |
| Epilepsy | 2 (0) | 0 (0) | 0.543 | 1 (0) | 0 (0) | 1.000 |
