# Supplementary material for: Propofol-based total intravenous anesthesia did not improve survival compared to desflurane anesthesia in breast cancer surgery
Source: PLoS One. 2019 Nov 7;14(11):e0224728. doi: 10.1371/journal.pone.0224728 (PMC6837387; doi:10.1371/journal.pone.0224728)
Supplement: S2 Table — (DOCX) [file pone.0224728.s002.docx]

**S2 Table**. Distribution of anesthesiologist and risk of mortality for overall patients and matched patients after propensity scoring

|  | **Overall patients** | | | | |  | **Matched patients** | | | | |
| --- | --- | --- | --- | --- | --- | --- | --- | --- | --- | --- | --- |
|  | **Desflurane**  N = 632 | **Propofol**  N = 344 | ***p*-value** | **Crude-HR (95% CI)** | ***p*-value** |  | **Desflurane**  N = 592 | **Propofol**  N = 296 | ***p*-value** | **Crude-HR (95% CI)** | ***p*-value** |
| Anesthesiologist |  |  | **< 0.001** |  | **0.618** |  |  |  | **< 0.001** |  | **0.785** |
| A | 7 (1) | 176 (51) |  | 1.00 |  |  | 7 (1) | 153 (52) |  | 1.00 |  |
| B | 64 (10) | 2 (1) |  | 1.04 (0.37-2.91) | 0.945 |  | 61 (10) | 2 (1) |  | 0.96 (0.34-2.70) | 0.940 |
| C | 41 (7) | 42 (12) |  | 1.18 (0.47-2.96) | 0.722 |  | 34 (6) | 36 (12) |  | 1.06 (0.40-2.80) | 0.902 |
| D | 12 (2) | 1 (0) |  | 1.68 (0.37-7.66) | 0.503 |  | 12 (2) | 1 (0) |  | 1.47 (0.32-6.73) | 0.617 |
| E | 71 (11) | 4 (1) |  | 0.52 (0.15-1.82) | 0.306 |  | 64 (11) | 3 (1) |  | 0.52 (0.15-1.81) | 0.301 |
| F | 79 (13) | 0 (0) |  | 0.18 (0.02-1.34) | 0.093 |  | 76 (13) | 0 (0) |  | 0.16 (0.02-1.21) | 0.076 |
| G | 106 (17) | 6 (2) |  | 0.74 (0.28-1.93) | 0.533 |  | 98 (17) | 4 (1) |  | 0.71 (0.27-1.88) | 0.493 |
| H | 80 (13) | 6 (2) |  | 0.55 (1.55-1.92) | 0.345 |  | 76 (13) | 5 (2) |  | 0.50 (0.14-1.77) | 0.283 |
| I | 40 (6) | 4 (1) |  | 0.58 (0.13-2.59) | 0.477 |  | 38 (6) | 3 (1) |  | 0.54 (0.12-2.40) | 0.417 |
| J | 9 (1) | 54 (16) |  | 1.00 (0.33-3.07) | 0.997 |  | 9 (2) | 44 (15) |  | 1.03 (0.34-3.18) | 0.953 |
| K | 41 (7) | 7 (2) |  | 0.30 (0.04-2.31) | 0.249 |  | 40 (7) | 6 (2) |  | 0.28 (0.04-2.11) | 0.214 |
| L | 44 (7) | 26 (8) |  | 1.59 (0.66-3.84) | 0.302 |  | 41 (7) | 24 (8) |  | 1.11 (0.42-2.92) | 0.833 |
| M | 28 (4) | 9 (3) |  | 1.44 (0.47-4.45) | 0.521 |  | 27 (5) | 9 (3) |  | 1.44 (0.47-4.45) | 0.521 |
| Others | 10 (2) | 7 (2) |  | 0.84 (0.11-6.43) | 0.868 |  | 10 (2) | 7 (2) |  | 0.84 (0.11-6.43) | 0.868 |

Those Anesthesiologists who have carried out less than 10 cases were grouped as others.
