# Supplementary material for: Propofol-based total intravenous anesthesia did not improve survival compared to desflurane anesthesia in breast cancer surgery
Source: PLoS One. 2019 Nov 7;14(11):e0224728. doi: 10.1371/journal.pone.0224728 (PMC6837387; doi:10.1371/journal.pone.0224728)
Supplement: S3 Table — (DOCX) [file pone.0224728.s003.docx]

**S3 Table.** Adjusted hazard ratio (HR) (95% CI) from the Cox regression proportional hazard survival models including anesthesiologists for overall patients (N = 976) and the matched patients (N = 888)

|  | **Overall patients (N = 976)** | |  | **Matched patients (N = 888)** | |
| --- | --- | --- | --- | --- | --- |
|  | **Adj-HR (95% CI)** | ***p*-value** |  | **Adj-HR (95% CI)** | ***p*-value** |
| Anesthesia, propofol (ref: Desflurane) | 0.70 (0.31–1.56) | 0.383 |  | 0.67 (0.28–1.61) | 0.369 |
| Age (yr) (ref: < 40) |  |  |  |  |  |
| 40-49 | 0.48 (0.22–1.06) | 0.068 |  | 0.62 (0.27–1.42) | 0.256 |
| 50-59 | 0.56 (0.26–1.22) | 0.144 |  | 0.62 (0.28–1.39) | 0.248 |
| 60-69 | 0.17 (0.05–0.58) | 0.005 |  | 0.25 (0.07–0.86) | 0.028 |
| ≥ 70 | 0.41 (0.12–1.43) | 0.161 |  | 0.42 (0.11–1.65) | 0.213 |
| ASA (ref: I) |  |  |  |  |  |
| II | 0.79 (0.40–1.58) | 0.507 |  | 0.84 (0.41–1.74) | 0.642 |
| III | 3.76 (1.44–9.80) | 0.007 |  | 4.46 (1.69–11.8) | 0.002 |
| TNM Stage of primary tumor, II+III (ref: 0+I) | 7.07 (3.03–16.5) | < 0.001 |  | 6.31 (2.73–14.6) | < 0.001 |
| ER (ref: negative) | 0.69 (0.36-1.32) | 0.263 |  | 0.73 (0.37-1.44) | 0.361 |
| PR (ref: negative) | 1.19 (0.61–2.33) | 0.603 |  | 1.20 (0.60-2.43) | 0.606 |
| Neoadjuvant chemotherapy (ref: no) | 11.9 (5.72–24.8) | < 0.001 |  | 13.2 (6.13–28.4) | < 0.001 |
| Intraoperative NSAIDs (ref: no) |  |  |  | 0.83 (0.30–2.35) | 0.730 |
| Adjuvant chemotherapy (ref: no) | 0.73 (0.32–1.69) | 0.462 |  | 0.84 (0.36–1.98) | 0.688 |
| Adjuvant hormonal therapy (ref: no) | 0.43 (0.23–0.80) | 0.008 |  | 0.43 (0.22–0.83) | 0.012 |

All multivariable HRs were adjusted by those variables significant (*p* < 0.05) in the univariable analyses and anesthesiologists (n = 20) simultaneously except anesthesia.

BMI = body mass index; ASA = American Society of Anesthesiologists; TNM = tumor-node-metastasis; CCI = Charlson comorbidity index; MET = metabolic equivalents; NSAID = nonsteroidal anti-inflammatory drugs; ER = estrogen receptor; PR = progesterone receptor; TNBC = triple-negative breast cancer.
